# The impact of an immersive digital therapeutic tool on experimental pain: a pilot randomized within-subject experiment with an active control condition

**DOI:** 10.3389/fpain.2024.1366892

**Published:** 2024-06-06

**Authors:** Sanoussy Diallo, Serge Marchand, Alexandre Dumais, Stéphane Potvin

**Affiliations:** ^1^ Centre de Recherche de l’Institut Universitaire en Santé Mentale de Montréal, Montreal, QC, Canada; ^2^ Department of Psychiatry and Addiction, Faculty of Medicine, University of Montreal, Montreal, QC, Canada; ^3^ Department of Surgery, Faculty of Medicine, University of Sherbrooke, Sherbrooke, QC, Canada; ^4^ Institut National de Psychiatrie Légale Philippe-Pinel, Montreal, QC, Canada

**Keywords:** pain, virtual reality, emotion, within-subject design, psychophysical

## Abstract

**Background:**

Pain is a complex and multifaced sensory and emotional experience. Virtual reality (VR) has shown promise in reducing experimental pain and chronic pain. This study examines an immersive VR environment initially designed for endometriosis patients, which demonstrated short-term analgesic effects. The research aims to determine the impact of the VR environment on experimental pain intensity and unpleasantness both during and after VR exposure (3D with binaural beats), while using an active control condition (2D with no binaural beats). Additionally, a secondary objective of the study was to identify the psychological and psychophysical factors that predict the analgesic effects of the immersive digital therapeutic tool.

**Methods:**

The study involved twenty-one healthy individuals and used a within-subject design, comparing a VR treatment with an active control condition. Continuous heat stimulation was applied to the left forearm with a Peltier thermode. Pain ratings were collected for immediate and short-term effects.

**Results:**

In both the VR and Control conditions, there were no significant differences in pain intensity before, during, and after exposure. However, during VR exposure, there was a significant decrease in pain unpleasantness as compared to before exposure (*p* < 0.001), with a 27.2% pain reduction. In the Control condition, there were no significant differences in pain unpleasantness during and after exposure. Furthermore, no psychological and psychophysical factors predicted the analgesic effects.

**Discussion:**

The study investigated how a VR environment affected experimentally induced pain in healthy volunteers. It showed that VR reduced pain unpleasantness during exposure but had no lasting impact. The VR environment mainly influenced the emotional aspect of pain, possibly due to its inclusion of binaural beats and natural stimuli. The study suggests that the VR environment should be tested in chronic pain population with high distress levels.

**Registration number (clinicaltrials.gov):**

NCT06130267.

## 1 Introduction

Pain is shaped by the context, meaning and the individual's psychological state (1). While the sensory component of pain (e.g., its intensity) refers to the physical sensation evoked by tissue damage or injury, the emotional component (e.g., its unpleasantness) is equally important and can significantly influence a person's pain experience (2). Most people recover from pain following an injury or operation and return to normalcy. However, there are situations when the pain lasts longer or appears suddenly without any prior history of an accident or operation. Pain that persists for more than 12 weeks despite medication or treatment is referred to as chronic or short-term pain (3). In terms of pharmacological treatment, several classes of medications have demonstrated efficacy in relieving pain, including antidepressants, certain anticonvulsants, opioids and non-steroidal anti-inflammatory drugs (4–7). However, the efficacy of some of these treatments is mixed (anticonvulsants), and some have potentially serious side effects (opioids). Among non-pharmacological pain treatments, virtual reality (VR) is one of the modalities that has attracted the most research interest over the past decade. Its potential efficacy has been tested in a wide range of painful conditions, from post-operative pain and neuropathic pain to pediatric pain and pain associated with medical procedures (4–7). To date, most clinical trials have included small samples and the proposed interventions have generally been offered over short periods of time. Despite these limitations, virtual reality has produced highly promising results, especially in the case of immediate pain (8), certainly justifying further work in this area.

To gain a better understanding of the potential mechanisms of action of virtual reality, several experimental studies have been carried out in healthy volunteers, using psychophysical procedures to administer nociceptive stimuli in a controlled manner. Regardless of the modality used (thermal, mechanical stimulation, etc.), the vast majority of studies to date have shown that virtual reality produces analgesic effects in a laboratory setting (9–12). Although promising, these studies have significant limitations. In most cases, they have focused on pain intensity, without addressing the unpleasantness of pain (13–15). More importantly, studies in the field often lack of an active control condition (i.e., non-immersive VR) (16). Indeed, in most studies, the control condition was a simple baseline condition where participants were asked to look at a blank screen (9, 11, 17). This is an important limitation which makes it difficult to properly interpret findings considering that such studies cannot rule out the possibility that simply wearing a head mounted display (HMD; with no VR content) may be sufficiently distracting to produce analgesic effects. It is important to point out, however, that a few studies have used minimal control conditions (example: HMD showing a black screen) and have shown that VR environments produce greater analgesic effects (18).

For this pilot study, we used a VR environment that was initially developed for the treatment of endometriosis, a chronic inflammatory disease defined by the presence of endometrial tissue outside the uterus, causing pelvic pain and infertility (19, 20). This VR environment, known as Endocare, consists in a session during which patients are immersed in a playful, calming environment (e.g., a landscape consisting of water and a beach). During the session, the background sound is made up, among other things, of binaural beats, for which the relaxing effects have been demonstrated (21). As compared to other VR interventions, the tool was not designed to primarily produce attention-grasping effects. The efficacy of this VR environment has been evaluated in a randomized controlled trial of endometriosis patients (22). The analgesic effects persisted for 4 h after administration. As such, the persistency of analgesic effects suggests that the VR environment recruits endogenous pain inhibition mechanisms. This echoes the results of a recent experimental study (23) which tested a VR environment on 38 volunteers and found that it was possible to directly enhance the endogenous pain inhibition efficacy in healthy volunteers.

Several psychological factors are commonly associated with pain across different pathologies, and some studies showed that we can predict treatment response with these variables in both clinical and non-clinical populations (24–26). In fact, according to a meta-analysis, 16 studies involving healthy volunteers reported a predictive relationship between psychological factors and experimental pain, while seven showed variable results (27). Such psychological factors include pain catastrophizing, sleep quality, anxiety, the severity of the pain and its impact on functioning (28, 29). A systematic review (30) on the use of VR as a distraction technology emphasized the role of several psychological aspects in the efficacy of analgesic distraction. The sense of presence is another key element influencing the efficacy of VR as diversion techniques (31, 32), with greater sense of presence being generally associated with greater treatment response. Finally, in the search of individual characteristics predicting treatment response in the field of pain, psychophysical procedures are growingly used. Thus far, it has been shown that one of the best predictors of pain outcomes is the efficacy of endogenous pain inhibition mechanisms including inhibitory conditioned pain modulation (33, 34).

To gain a better understanding of the mechanisms involved in a novel immersive digital tool that was developed for the treatment of endometriosis, we carried out an experimental pilot study in healthy volunteers. A randomized cross-over design was adopted, and pain was elicited using tonic thermal noxious stimuli. Considering that experimental studies that have tested VR interventions have focused mostly on pain intensity, and that control conditions have been inadequate in most cases, the current pilot study measured pain unpleasantness and used an active control condition. More precisely, we aimed to compare a 3D environment with sound (e.g., binaural beats) to a 2D environment with no sounds, both projected in a VR headset. Since the novel tool appears to produce clinical benefits that persist over time, pain outcomes were measured both *during* and *after* the administration of the VR environment. A secondary objective of the study was to identify the psychological and psychophysical factors that predict the analgesic effects of the immersive digital therapeutic tool.

## 2 Methods

### 2.1 Participants

The selection criteria were healthy men and women between 18 and 50 years old who were willing to participate in the study and signed the informed consent form. The exclusion criteria were if a person presented a (i) neurological disorder, (ii) substance use disorder, (iii) severe mental health disorder, (iv) chronic pain (e.g., pain lasting longer than 12 weeks), (v) any acute and unstable medical condition, and (vi) taking medication that acts on the central nervous system.

### 2.2 Ethics approval

This pilot study was conducted in compliance with good clinical practice guidelines, the principles of the Declaration of Helsinki and Health Canadian laws and regulation. It was reviewed and approved on June 3rd, 2022, by the CIUSSS de l'Est-de-l'île-de-Montréal. All participants completed and signed the informed consent form before inclusion in the study and before any study-related procedure began.

### 2.3 Clinical evaluation

The severity of transient immediate pain was assessed with the *Brief Pain Inventory* (BPI) (35). The BPI is a self-administered measure of the sensory and reactive dimensions of pain that is the severity or intensity of the pain and the level of interference it has on various aspects of life. The internal consistency of the BPI has been reported to range between 0.87 and 0.92 while the test-retest reliability falls between 0.86 and 0.97 (36, 37). Since the BPI was developed for the assessment of chronic pain, here, we only used Item 3 on the worst pain intensity experienced in the last week. The overall sleep quality was evaluated with the *Pittsburgh Sleep Quality Index* (PSQI) (38). Each of the questionnaire's 19 self-reported items belongs to one of seven subcategories: subjective sleep quality, sleep latency, sleep duration, habitual sleep efficiency, sleep disturbances, use of sleeping medication, and daytime dysfunction. The PSQI has shown internal consistency ranging from 0.70 to 0.83 and the test-retest reliability between 0.72 and 0.90 (39, 40). The *Pain Catastrophizing Scale* (PCS) (41) was also administered. The PCS instructions ask to reflect on past painful experiences, and to indicate the degree to which they experienced each of 13 thoughts or feelings when experiencing pain, on 5-point scales with the end points (0) not at all and (4) all the time. The PCS demonstrates strong internal consistency, with reported values ranging between 0.84 and 0.97, and a test-retest reliability of approximately 0.75 (42). The *State-Trait Anxiety Inventory* (STAI) was also used (43, 44). It is a questionnaire composed of a 20 items scale designed to measure the state of anxiety. The STAI exhibits good internal consistency between 0.89 and 0.91 and the test-retest reliability between 0.70 and 0.88 (44, 45). Finally, the sense of presence experienced in a virtual environment was measured with the *Igroup Presence Questionnaire* (IPQ) (46). The IPQ has three subscales (1. Spatial Presence, 2. Involvement and 3. Experienced Realism) and one general item not belonging to a subscale (Sense of being there). The internal consistency and test-retest reliability for the IPQ are 0.87 and 0.74 (47).

### 2.4 Design and settings

This pilot study was a randomized within-subject design comparing the effect of the VR environment and an active Control condition. To mitigate carry-over effects, a 30-min rest period separated the administration of experimental and control conditions, a protocol implemented with precision based on our own experience (48). The order of conditions was counterbalanced (and randomized) for each participant to ensure robustness in the study design. The study was conducted between June 2022 and April 2023 at the research center of the *Institut Universitaire en Santé Mentale de Montréal*. Each participant dedicated approximately 2.5 h to the study and received appropriate incentives as acknowledgment for their valuable time.

#### 2.4.1 Immediate analgesic effect

A continuous heat stimulation was administered with a 3 cm^2^ Peltier-type thermode (Medoc Advanced Medical Systems, Ramat Yishay, Israel) and was applied for 2 min on different locations of the left forearm of participants for each stimulation. The thermode is a small heating plate connected to a computer, enabling precise and safe control of the temperatures used. The tonic heat pain stimulation was administered *before (test 1)* the experimental and control conditions, as well as *during (test 2)* both conditions and immediately after (*test 3*). In all cases, the experimental temperature reached a pre-determined fixed value and remained constant during the 2-min testing period. It was set at a value inducing a moderate level of pain during the pre-test.

During the pre-test, pain threshold and pain tolerance were first measured, and the participants were not asked to rate the pain unpleasantness. The experimental temperature started at 32 °C and gradually increased at a rate of 0.3 °C per second. We asked participants to subjectively report the point at which the sensation of heat began to be perceived as pain (pain threshold), as well as the point at which the pain became intolerable (tolerance). For each participant, we noted the temperature that caused a moderate pain level of 50 (T50) on a scale from 0 (no pain) to 100 (intolerable pain) by using a Computerized Visual Analogue Scale. The pre-test was repeated 3 times to obtain the most reliable measure of pain threshold, pain tolerance and T50.

The T50 was the temperature used for the administration of the 2-min tonic heat pain stimulations that were administered *before (test 1)*, *during (test 2)* both the experimental and control conditions and immediately after (test 3). Importantly, participants were not made aware that the *same* experimental temperature was used throughout the experiments. Also noteworthy, the tonic heat pain stimulations were only administered in the final minutes of both the experimental conditions in order to let the participants fully engage in the immersive experience before receiving the nociceptive inputs. At the end of each tonic heat pain stimulation, subjects verbally rated their pain intensity and their pain unpleasantness using a numerical rating scale, also ranging from 0 (no pain) to 100 (most intense pain tolerable).

#### 2.4.2 Short-term effect

**Figure 1:**
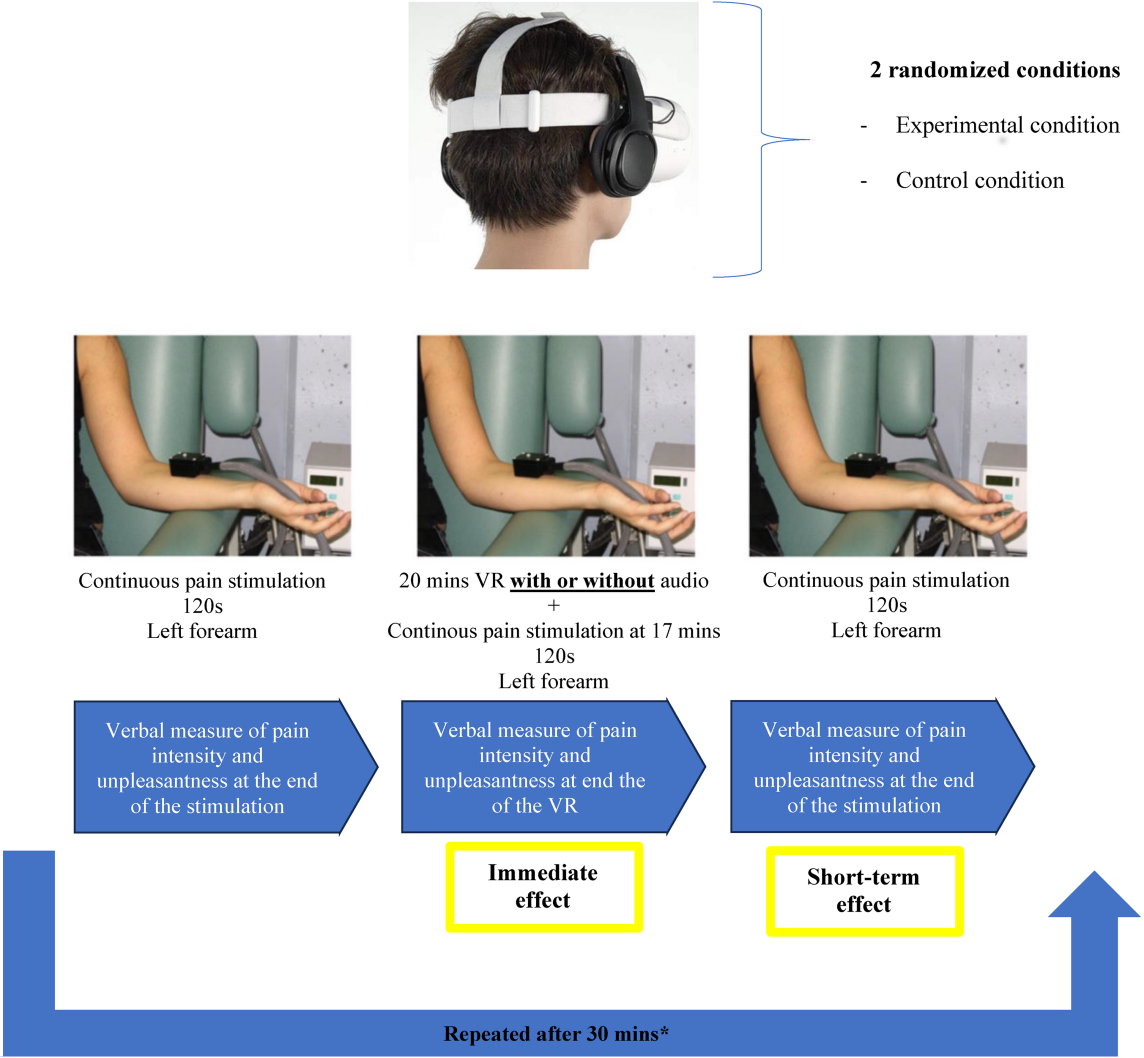
Study design. Tonic heat pain intensity and unpleasantness were measured before, during and after the Virtual Reality (VR) or Control conditions. The order the VR and Control conditions was counter balanced and randomized for each participant.

To measure the potential short-term effect of both the experimental and control conditions, we administered the same tonic heat pain stimulation immediately at the end of both conditions. Here again, subjects were asked to verbally rate their pain intensity and their pain unpleasantness using a numerical rating scale, also ranging from 0 (no pain) to 100 (most intense pain tolerable). For more information on the study design, please refer to Figure 1.

### 2.5 Treatment and control conditions description

**Figure 2:**
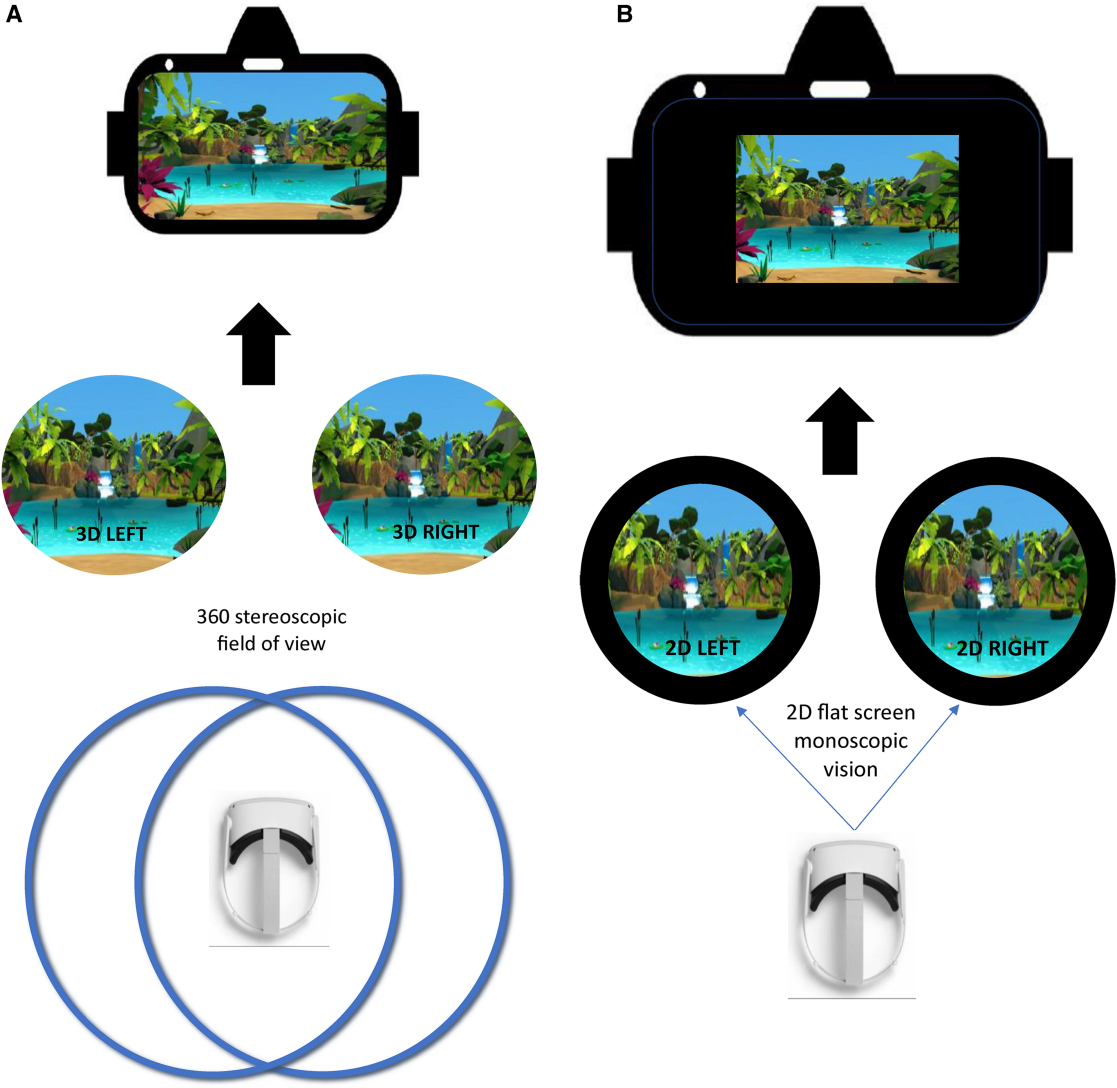
(**A**) Virtual reality (VR) environment. In this figure, you can see a 3D stereoscopic display, which uses two cameras to capture left and right images or video frames simultaneously. These images, viewed separately by each eye, mimic human vision. In VR, stereoscopic videos create depth perception, though there are only two viewpoints. (**B**) Control condition. In this figure, you can see a 2D monoscopic display, which shows the same image to both eyes simultaneously. It lacks depth perception and appears flat, hence the black background surrounding the image, whether it is regular 2D or an immersive 360/180 content.

The treatment was displayed through a VR headset (Oculus Quest 2) with a high-quality headphone (Audio-Technica ATH-M50x). This potential treatment is a standalone medical software device comprised of an application stored in a VR headset that is intended to mitigate pain (22) (see Figure 2A). It offers a 20-min treatment consisting of a combination of auditory (e.g., alpha/theta binaural beats, nature-based sounds) and visual (e.g., bilateral alternative simulations consisting of a sphere appearing and moving on a horizontal axis) therapeutic procedures integrated in a 3D-VR environment. Furthermore, the treatment was designed to guide participants to relax, to passively look at the environment, and to passively listen to the auditory components. The treatment involved no active interaction with the VR environment.

The control condition was displayed through a VR headset with a high-quality headphone (Audio-Technica ATH-M50x). This comparator was a 20-min control with the same composition as the experimental treatment (same context, environment and duration) but in 2D with 3 Degrees of Freedom and without any auditory stimulus (e.g., alpha/theta binaural beats, nature-based sounds) (see Figure 2B). Moreover, in this condition, there was no instructions provided to the participant.

### 2.6 Inhibitory conditioned pain modulation (iCPM)

The efficacy of inhibitory CPM was measured in a separate experimental session ∼one week apart from the VR session. To measure the inhibitory CPM, a continuous heat simulation was administered with a thermode for 2 min on the left forearm of participants. To capture the effects of inhibitory CPM, the test stimulus was administered twice, separated by the administration of the cold-pressor test (CPT) as the conditioning stimulus. By measuring pain elicited by the test stimulus before and after the conditioning stimulus, it was possible to measure iCPM (for more information, please refer to Supplementary Material).

### 2.7 Statistics

To test the immediate and short-term analgesic effect of the immersive digital therapeutic tool (primary objectives) and its superiority to the control condition, we used repeated-measures analyses of variance (ANOVA) with pain intensity and pain unpleasantness as the dependent variables. Given that the current study is a pilot trial, we did not perform a two-way repeated measures ANOVA. Instead, we performed repeated-measures ANOVAs on the VR and control conditions separately. For significant time effects, *post hoc* paired t-tests were performed, and were limited to the During vs. Before and After vs. Before comparisons. The magnitude of the analgesic effect of the VR environment was determined using Cohen's d (49).

To identify the predictors of response to the digital immersive therapeutic tool, Pearson's correlational analyses were performed between to determine whether the level of analgesia induced by the VR treatment and psychological variables (sense of presence, clinical pain, pain dramatization, anxiety and sleep quality) as well as the efficacy of inhibitory CPM. Due to the nature of the current trial, we adopted an uncorrected *p*-value, which was set at *p* < 0.05 for all analyses.

## 3 Results

### 3.1 Study participants

Twenty-three participants were recruited. The size of the sample was a pragmatic choice based on practical issues (e.g., time constraints). Two of them were excluded due to technical problems during the experiments and missing data. Twenty-one participants completed the full experiment; 17 were females and 13 were Caucasian. The mean age of participants was 23.2 years ±3.5, with 17.2 years of education on average and a mean of 5.7 ± 2.4 for the PSQI. In this sample, the mean of the BPI (Item 3) was 3.4 ± 2.1 and 3.2 ± 0.68 on the IPQ. For the STAI, the mean was 33.8 ± 9.5 and the mean of the pain catastrophizing was 19.1 ± 8.1. The mean of the stimulus test (T50) was 46.3 °C (SD = 1.5). The mean of the pain threshold was 43.3 °C (SD = 4.0) and the mean of the pain tolerance (maximum pain) was 47.7 °C (SD = 1.7).

#### 3.1.1 VR condition

##### 3.1.1.1 Intensity

**Figure 3:**
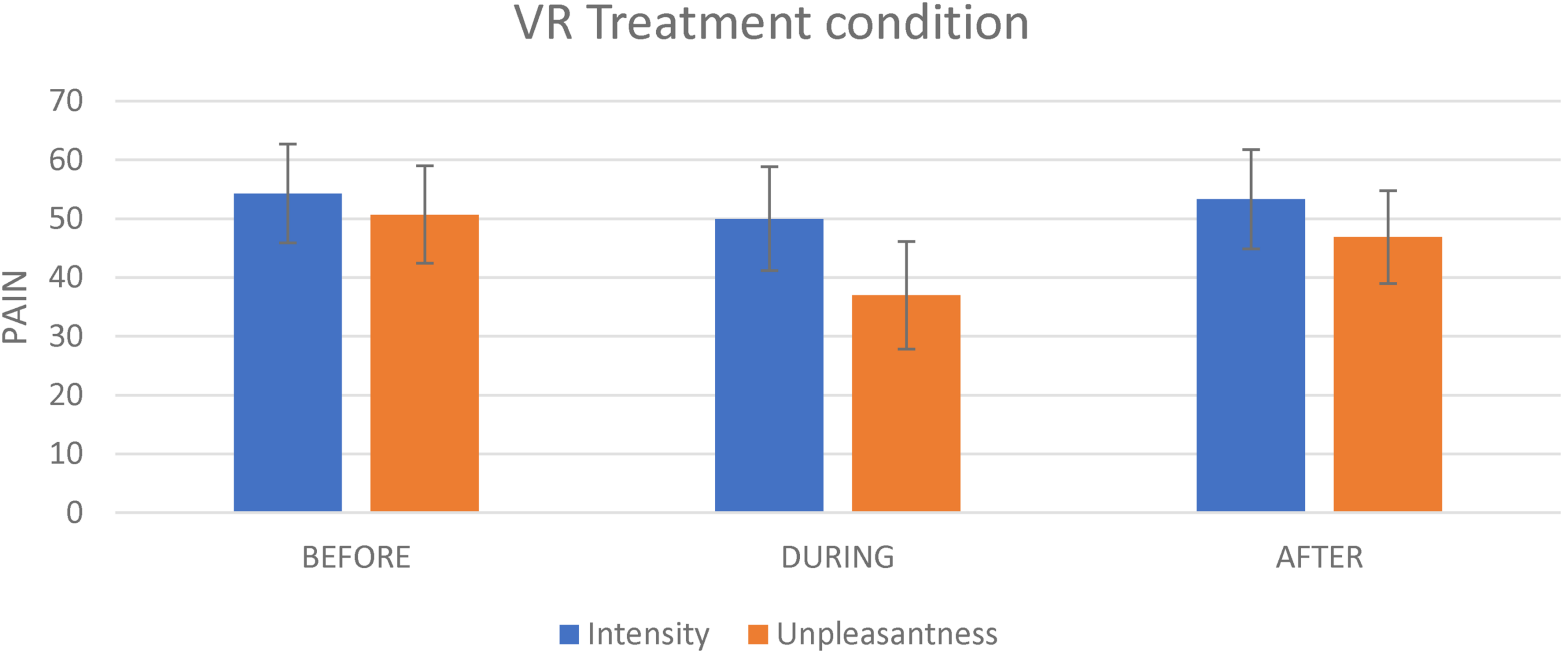
The effect of the experimental condition on pain intensity and pain unpleasantness. This figure shows the effect of the virtual reality on pain intensity and on pain unpleasantness evoked by the tonic thermal noxious stimuli.

For the VR condition, the mean pain intensity evoked by the tonic nociceptive stimulation (i.e., the thermode) before, during and after VR exposure was respectively 54.3 (SD = 19.6), 50.0 (SD = 20.7) and 53.3 (SD = 19.8). The repeated-measures ANOVA revealed no significant of time [F(2,40) = 1.2; *p* = 0.313] (see Figure 3).

##### 3.1.1.2 Pain unpleasantness

For the VR treatment, the mean unpleasantness evoked by the nociceptive stimulation (i.e., the thermode) before, during and After VR exposure was respectively 50.7 (SD = 19.3), 36.9 (SD = 21.4), and 46.9 (SD = 18.5). The repeated measures analyses revealed a significant effect of time [F(2,40) = 8.3; *p* = 0.001]. There was a significant decrease in pain unpleasantness during VR exposure as compared to Before exposure (*t* = 4.2; *p* = 0.001; Cohen's d = 0.7; 27.2% change) (see Figure 3). By contrast, there was no significant difference for the After vs. Before comparison (*t* = 1.0; *p* = 0.335).

#### 3.1.2 Control condition

##### 3.1.2.1 Intensity

**Figure 4:**
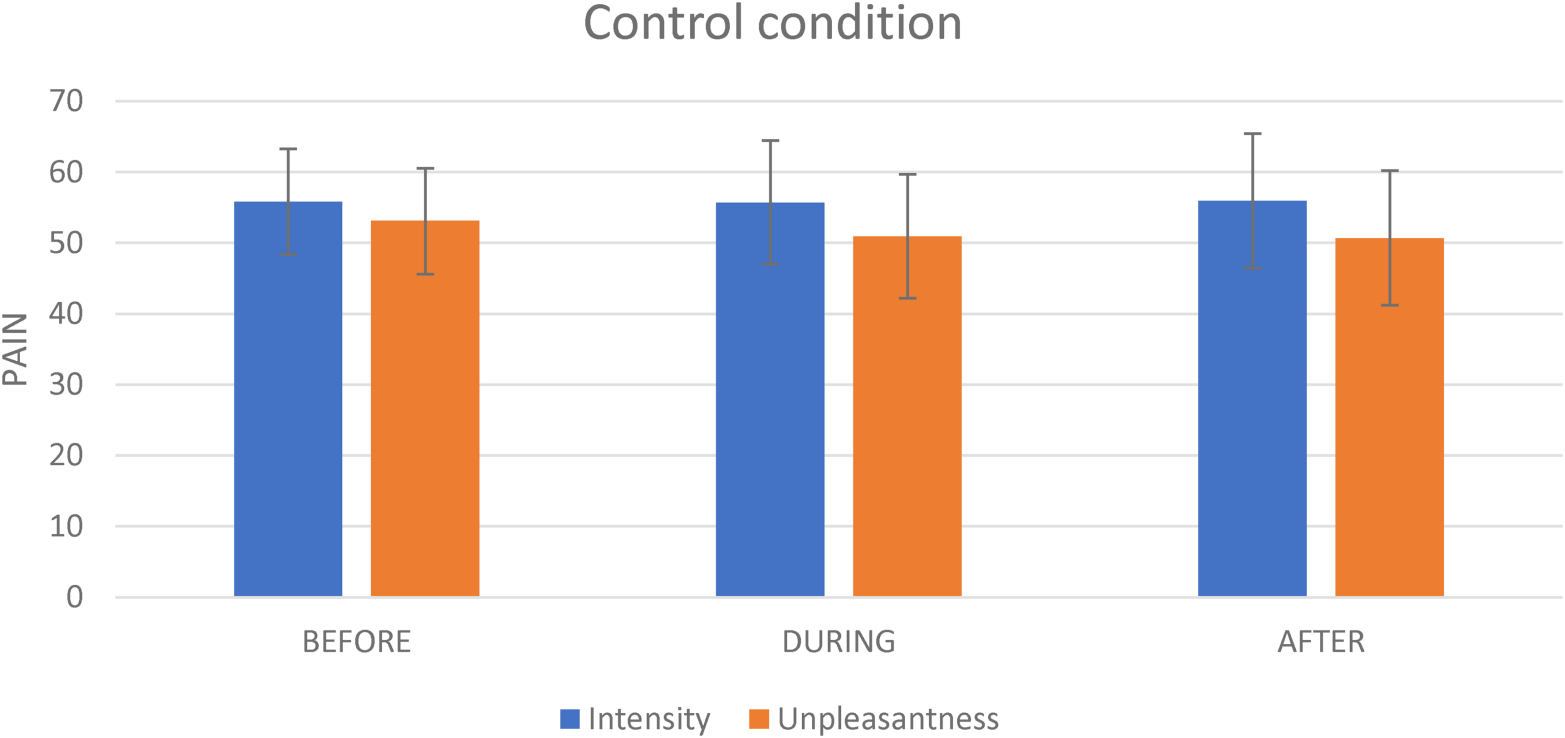
The effect of the control condition on pain intensity and pain unpleasantness. This figure shows the effect of the virtual reality on pain intensity and on pain unpleasantness evoked by the tonic thermal noxious stimuli.

For the Control condition, the mean pain intensity evoked by the nociceptive stimulation (i.e., the thermode) before, during and after the Control condition administration was respectively 55.8 (SD = 17.5), 55.7 (SD = 20.5) and 55.9 (SD = 22.2). The repeated-measures analyses revealed no significant effect of time [F(2,40) = 0.002; *p* = 0.998] (see Figure 4).

##### 3.1.2.2 Pain unpleasantness

For the Control condition, the mean pain unpleasantness evoked by the nociceptive stimulation (i.e., the thermode) before, during and after the Control administration was respectively 53.1 (SD = 20.6), 50.9 (SD = 22.8) and 50.7 (SD = 23.3) (see Figure 4). The repeated-measures analyses revealed no significant effect of time [F(2,40) = 0.2; *p* = 0.817].

### 3.2 Difference in experimentally induced pain between the VR treatment and the control condition

To examine potential differences between the VR and control conditions, the change in pain unpleasantness (delta) was calculated for each participant for the During vs. Before comparison. In an explanatory manner, we then performed a paired t-test using these delta-values. This explanatory analysis showed a stronger effect of the VR condition over the Control condition on pain unpleasantness for the During vs. Before comparison (*t* = 2.4; *p* = 0.027).

### 3.3 Predictors of analgesic response

Pearson's correlation coefficients were computed to assess the linear relationships between psychological variable, inhibitory CPM efficacy and the analgesic effect of the VR treatment. Analyses were restricted to pain unpleasantness since the VR environment did not significantly impact pain intensity. The correlations between the clinical pain (BPI, Item 3; *r* = 0.315; *p* = 0.165), pain catastrophizing (PCS; *r* = 0.177, *p* = 0.442), anxiety (STAI; *r* = 0.033; *p* = 0.888) and sleep (PSQI; *r* = −0.019; *p* = 0.241) with the analgesic effect of the VR treatment were not significant. Likewise, the sense of presence (IPQ) showed no significant relation (*r* = −0.236; *p* = 0.303) with the analgesic effect of the VR treatment. Finally, the correlation between the efficacy of inhibitory CPM and the analgesic efficacy of the VR treatment showed no significant correlation (*r* = 0.010; *p* = 0.965).

## 4 Discussion

The main objective of the current experimental pilot study was to examine the effect of a VR environment on experimental pain in healthy volunteers to gain a better understanding of its mechanisms of action, while paying attention to both pain intensity and pain unpleasantness. Both the immediate and short-term effects of the VR environments were tested, and a minimally active control condition was used. Results showed an immediate effect of the VR environment on pain unpleasantness but no short-term effect. The analgesic efficacy of the VR environment on pain unpleasantness did not correlate with any of the psychological and physiological (e.g., iCPM) variables measured.

The main finding of the current study is that the VR environment produced a specific effect on the emotional component of pain, with no observed effect on its sensory-discriminative component. This finding is coherent with the evidence pointing to significant differences between these two pain components. First, there reliable is evidence that these two components are processed by different brain regions (50). Moreover, there is evidence demonstrating that pain intensity and unpleasantness may exhibit divergent responses to certain analgesic procedures such as transcutaneous electric nerve stimulation, hypnosis and opioids (51–55). One of the potential explanations for this specific effect on pain unpleasantness has to do with the fact that binaural beats are included in the immersive digital tool. Although there is no agreement upon the mechanisms underlying binaural auditory beats, there is a growing support for the claim that binaural auditory beats affect psychophysiological states. Indeed, numerous studies have reported that binaural beats exposure leads to a change in pain perception, autonomic measures and negative emotions, and is known to produce a relaxing effect (56–58). Another explanation would be the exposure to visual stimuli from nature (e.g., sand, water, trees, mountains) and nature-based sounds that are displayed in the VR environment. Multiple studies have shown that both nature-based auditory and visual stimuli can reduce stress and anxiety and produce pain relief (59–65). The specific effect on pain unpleasantness of the VR environment in the current study is relevant for the design of future trials using this immersive digital tool. It does suggest, indeed, that the VR environment should be primarily tested in chronic pain populations with significant levels of distress. While anxio-depressive symptoms are usually quite prevalent in chronic pain (66, 67), the comorbidity rates are even higher in chronic pain conditions with no clear physiological causes, such as functional pain syndromes (68).

Although this was not directly tested, the results observed here do not suggest that the VR environment activated endogenous pain inhibition mechanisms (e.g., iCPM). Indeed, no correlation was observed between the effect of the environment on pain unpleasantness and iCPM; moreover, these beneficial effects of the VR environment were only observed during the immediate administration, without these effects persisting after its interruption. As such, these results are inconsistent with the previous clinical study with the immersive digital tool which has highlighted, in endometriosis patients, analgesic effects that lasted up to 4 h after the environment administration (22). There are two possible explanations for this discrepancy in results between the two studies. On the one hand, the previous clinical study involved people with chronic pain, whereas the present study included healthy volunteers with no clinical pain. It is therefore possible that the effect of the immersive digital tool may differ depending on whether the pain is iatrogenic or experimentally induced. The other point to consider is that in the previous study, pain measurements were taken for a long period of time (4 h) after the VR environment was discontinued, whereas in the present study, we took only one measurement, immediately after the VR environment was discontinued. The limited duration of the after-treatment measurement may have prevented the observation of delayed analgesic effects. Significant improvements may have only been observable with longer time frames.

Contrary to our expectations, we did not observe any correlations between the analgesic effect of the VR environment and the various clinical variables measured in the project, namely anxiety, sub-clinical pain, pain catastrophizing, and sleep. As such, this lack of correlation is inconsistent with past studies conducted with clinical populations or healthy volunteers tested in experimental settings (69, 70). The main reason for this lack of correlation in the present study could be explained by the fact that our study included a non-clinical sample. The recruited sample presented rather low levels of anxiety, sub-clinical pain, pain catastrophizing and sleep problems as compared to normative and clinical samples (35, 38, 41, 43, 44, 46).

One of the strengths of this pilot study is that the control condition was immersive in that participants had to wear the VR headset during this condition. This procedure markedly contrasts with many past studies in the field that used simple baseline conditions (63, 71, 72). The use of a minimally active control condition in the current experimental study is important in that it allowed to control for mere effects of wearing a VR headset. Despite this important methodological strength, the present study had some limitations. As mentioned previously, one of the limitations of the current study is that only one pain measurement was taken immediately at the end of the administration of the experimental and control conditions. Another limitation is the sample size (*N* = 21), which was relatively small. Small samples are associated with decreased statistical power. This increases the likelihood of committing type II errors, where smaller effects are not detected due to insufficient data. The inclusion of a small number of participants also makes populational inferences more uncertain. Finally, we acknowledge that the use of the BPI for measuring transient painful somatic symptoms is a study limitation, since the BPI was originally developed for the measurement of chronic pain (33), which was an exclusion criterion in the current study. Furthermore, another limitation is that we did not measure the IPQ for the control condition; thus, we cannot determine whether the observed difference between the VR and control conditions could be attributed to differences in the feelings of presence elicited by both conditions.

In a pilot randomized-controlled study examining the potential effects of a novel VR environment on experimentally induced pain using an active control condition, our results showed that the digital immersive tool produced significant immediate effects on pain unpleasantness that did not last at the end of the VR stimulation. With the positive results obtained in the present project, one of the next steps would be to investigate in experimental settings the relative contribution of the digital tool components to the analgesic effects observed, namely the binaural sounds, the nature-based sounds, and/or the visual stimuli of nature scenes. Another avenue would be to test the analgesic effects of the VR treatment in larger samples of participants, especially clinical populations living with chronic pain who also experience significant levels of anxio-depressive symptoms. Finally, considering the fact that our VR environment does not engage higher cognitive functions (e.g., decision-making) like other VR environments that have been used in past studies (e.g., videogames) (73–75), it would be of interest to perform head-to-comparisons of both approaches in order to determine if their analgesic effets and mechanisms of action are comparable or not.

## Data Availability

The data that support the findings of this study are available upon reasonable request from the corresponding author, SP, but are only redistributable to researchers engaged in IRB approved research collaborations. Requests to access the datasets should be directed to stephane.potvin@umontreal.ca.

## References

[1] Raja SN Carr DB Cohen M Finnerup NB Flor H Gibson S The revised international association for the study of pain definition of pain: concepts, challenges, and compromises. Pain. (2020) 161(9):1976–82. 10.1097/j.pain.0000000000001939 32694387 PMC7680716

[2] Lumley MA Cohen JL Borszcz GS Cano A Radcliffe AM Porter LS Pain and emotion: a biopsychosocial review of recent research. J Clin Psychol. (2011) 67(9):942–68. 10.1002/jclp.20816 21647882 PMC3152687

[3] Treede R-D Rief W Barke A Aziz Q Bennett MI Benoliel R Chronic pain as a symptom or a disease: the IASP classification of chronic pain for the international classification of diseases (ICD-11). Pain. (2019) 160(1):19–27. 10.1097/j.pain.0000000000001384 30586067

[4] Asrar MM Kumari S Sekhar BC Bhansali A Bansal D. Relative efficacy and safety of pharmacotherapeutic interventions for diabetic peripheral neuropathy: a systematic review and Bayesian network meta-analysis. Pain Physician. (2021) 24(1):E1–e14. 33400429

[5] Busse JW Sadeghirad B Oparin Y Chen E Goshua A May C Management of acute pain from non-low back, musculoskeletal injuries: a systematic review and network meta-analysis of randomized trials. Ann Intern Med. (2020) 173(9):730–8. 10.7326/M19-3601 32805127

[6] Ferreira GE McLachlan AJ Lin CC Zadro JR Abdel-Shaheed C O'Keeffe M Efficacy and safety of antidepressants for the treatment of back pain and osteoarthritis: systematic review and meta-analysis. Br Med J. (2021) 372:m4825. 10.1136/bmj.m4825 33472813 PMC8489297

[7] Migliorini F Maffulli N Baroncini A Eschweiler J Tingart M Quack V. Opioids for chronic low back pain management: a Bayesian network meta-analysis. Expert Rev Clin Pharmacol. (2021) 14(5):635–41. 10.1080/17512433.2021.1903316 33706636

[8] Mallari B Spaeth EK Goh H Boyd BS. Virtual reality as an analgesic for acute and chronic pain in adults: a systematic review and meta-analysis. J Pain Res. (2019) 12:2053–85. 10.2147/JPR.S200498 31308733 PMC6613199

[9] Loreto-Quijada D Gutiérrez-Maldonado J Nieto R Gutiérrez-Martínez O Ferrer-García M Saldaña C Differential effects of two virtual reality interventions: distraction versus pain control. Cyberpsychol Behav Soc Netw. (2014) 17(6):353–8. 10.1089/cyber.2014.0057 24892197

[10] Hayashi K Aono S Shiro Y Ushida T. Effects of virtual reality-based exercise imagery on pain in healthy individuals. Biomed Res Int. (2019) 2019:5021914. 10.1155/2019/5021914 31119173 PMC6500693

[11] Hoffman HG Sharar SR Coda B Everett JJ Ciol M Richards T Manipulating presence influences the magnitude of virtual reality analgesia. Pain. (2004) 111(1-2):162–8. 10.1016/j.pain.2004.06.013 15327820

[12] Phelan I Furness PJ Fehily O Thompson AR Babiker NT Lamb MA A mixed-methods investigation into the acceptability, usability, and perceived effectiveness of active and passive virtual reality scenarios in managing pain under experimental conditions. J Burn Care Res. (2019) 40(1):85–90. 10.1093/jbcr/iry052 30247616

[13] Brea-Gómez B Torres-Sánchez I Ortiz-Rubio A Calvache-Mateo A Cabrera-Martos I López-López L Virtual reality in the treatment of adults with chronic low back pain: a systematic review and meta-analysis of randomized clinical trials. Int J Environ Res Public Health. (2021) 18(22):11806. 10.3390/ijerph182211806 34831562 PMC8621053

[14] Sharifpour S Manshaee G Sajjadian I. Effects of virtual reality therapy on perceived pain intensity, anxiety, catastrophising and self-efficacy among adolescents with cancer. Couns Psychother Res. (2020) 21. 10.1002/capr.12311

[15] Thong ISK Jensen MP Miró J Tan G. The validity of pain intensity measures: what do the NRS, VAS, VRS, and FPS-R measure? Scand J Pain. (2018) 18(1):99–107. 10.1515/sjpain-2018-0012 29794282

[16] Honzel E Murthi S Brawn-Cinani B Colloca G Kier C Varshney A Virtual reality, music, and pain: developing the premise for an interdisciplinary approach to pain management. Pain. (2019) 160(9):1909–19. 10.1097/j.pain.0000000000001539 30817437 PMC7279616

[17] Karaman D Taşdemir N. The effect of using virtual reality during breast biopsy on pain and anxiety: a randomized controlled trial. J Perianesth Nurs. (2021) 36(6):702–5. 10.1016/j.jopan.2021.04.007 34686402

[18] Law EF Dahlquist LM Sil S Weiss KE Herbert LJ Wohlheiter K Videogame distraction using virtual reality technology for children experiencing cold pressor pain: the role of cognitive processing. J Pediatr Psychol. (2010) 36(1):84–94. 10.1093/jpepsy/jsq063 20656761 PMC3107585

[19] Giudice LC. Clinical practice. Endometriosis. N Engl J Med. (2010) 362(25):2389–98. 10.1056/NEJMcp1000274 20573927 PMC3108065

[20] Vercellini P Buggio L Frattaruolo MP Borghi A Dridi D Somigliana E. Medical treatment of endometriosis-related pain. Best Pract Res Clin Obstet Gynaecol. (2018) 51:68–91. 10.1016/j.bpobgyn.2018.01.015 29530425

[21] Garcia-Argibay M Santed MA Reales JM. Efficacy of binaural auditory beats in cognition, anxiety, and pain perception: a meta-analysis. Psychol Res. (2019) 83(2):357–72. 10.1007/s00426-018-1066-8 30073406

[22] Merlot B Dispersyn G Husson Z Chanavaz-Lacheray I Dennis T Greco-Vuilloud J Pain reduction with an immersive digital therapeutic tool in women living with endometriosis-related pelvic pain: randomized controlled trial. J Med Internet Res. (2022) 24(9):e39531. 10.2196/39531 36129733 PMC9536521

[23] Mehesz E Karoui H Strutton PH Hughes SW. Exposure to an immersive virtual reality environment can modulate perceptual correlates of endogenous analgesia and central sensitization in healthy volunteers. J Pain. (2021) 22(6):707–14. 10.1016/j.jpain.2020.12.007 33465506

[24] Baum C Huber C Schneider R Lautenbacher S. Prediction of experimental pain sensitivity by attention to pain-related stimuli in healthy individuals. Percept Mot Skills. (2011) 112(3):926–46. 10.2466/02.09.22.PMS.112.3.926-946 21853779

[25] Oosterman JM Dijkerman HC Kessels RP Scherder EJ. A unique association between cognitive inhibition and pain sensitivity in healthy participants. Eur J Pain. (2010) 14(10):1046–50. 10.1016/j.ejpain.2010.04.004 20493746

[26] Edwards RR Campbell CM Fillingim RB. Catastrophizing and experimental pain sensitivity: only in vivo reports of catastrophic cognitions correlate with pain responses. J Pain. (2005) 6(5):338–9. 10.1016/j.jpain.2005.02.013 15890636

[27] Hansen MS Horjales-Araujo E Dahl JB. Associations between psychological variables and pain in experimental pain models. A systematic review. Acta Anaesthesiol Scand. (2015) 59(9):1094–102. 10.1111/aas.12555 26088747

[28] Linton SJ Shaw WS. Impact of psychological factors in the experience of pain. Phys Ther. (2011) 91(5):700–11. 10.2522/ptj.20100330 21451097

[29] Zhuo M. Neural mechanisms underlying anxiety–chronic pain interactions. Trends Neurosci. (2016) 39(3):136–45. 10.1016/j.tins.2016.01.006 26878750

[30] Stefano Triberti CR Riva G. Psychological factors influencing the effectiveness of virtual reality–based analgesia: a systematic review. Cyberpsychol Behavior Soc Networking. (2014) 17(6):335–45. 10.1089/cyber.2014.0054 24892195

[31] Riva G Mantovani F. From the body to the tools and back: a general framework for presence in mediated interactions⋆. Interact Comput. (2012) 24(4):203–10. 10.1016/j.intcom.2012.04.007

[32] Villani D Repetto C Cipresso P Riva G. May I experience more presence in doing the same thing in virtual reality than in reality? An answer from a simulated job interview. Interact Comput. (2012) 24:265–72. 10.1016/j.intcom.2012.04.008

[33] Wilder-Smith OH Schreyer T Scheffer GJ Arendt-Nielsen L. Patients with chronic pain after abdominal surgery show less preoperative endogenous pain inhibition and more postoperative hyperalgesia: a pilot study. J Pain Palliat Care Pharmacother. (2010) 24(2):119–28. 10.3109/15360281003706069 20504133

[34] Yarnitsky D. Role of endogenous pain modulation in chronic pain mechanisms and treatment. Pain. (2015) 156:S24–31. 10.1097/01.j.pain.0000460343.46847.58 25789433

[35] Cleeland CS Ryan KM. Pain assessment: global use of the brief pain inventory. Ann Acad Med Singap. (1994) 23(2):129–38. 8080219

[36] Atkinson TM Mendoza TR Sit L Passik S Scher HI Cleeland C The brief pain inventory and its “pain at its worst in the last 24 hours” item: clinical trial endpoint considerations. Pain Med. (2010) 11(3):337–46. 10.1111/j.1526-4637.2009.00774.x 20030743 PMC3806650

[37] Poundja J Fikretoglu D Guay S Brunet A. Validation of the French version of the brief pain inventory in Canadian veterans suffering from traumatic stress. J Pain Symptom Manage. (2007) 33(6):720–6. 10.1016/j.jpainsymman.2006.09.031 17531912

[38] Buysse DJ Reynolds CF 3rd Monk TH Berman SR Kupfer DJ. The Pittsburgh sleep quality index: a new instrument for psychiatric practice and research. Psychiatry Res. (1989) 28(2):193–213. 10.1016/0165-1781(89)90047-4 2748771

[39] Ait-Aoudia M Levy PP Bui E Insana S de Fouchier C Germain A Validation of the French version of the Pittsburgh sleep quality index addendum for posttraumatic stress disorder. Eur J Psychotraumatol. (2013) 4:129–38. 10.3402/ejpt.v4i0.19298 PMC3773169 24044071

[40] Mollayeva T Thurairajah P Burton K Mollayeva S Shapiro CM Colantonio A. The Pittsburgh sleep quality index as a screening tool for sleep dysfunction in clinical and non-clinical samples: a systematic review and meta-analysis. Sleep Med Rev. (2016) 25:52–73. 10.1016/j.smrv.2015.01.009 26163057

[41] Sullivan MJL Bishop SR Pivik J. The pain catastrophizing scale: development and validation. Psychol Assess. (1995) 7(4):524–32. 10.1037/1040-3590.7.4.524

[42] Ikemoto T Hayashi K Shiro Y Arai YC Marcuzzi A Costa D A systematic review of cross-cultural validation of the pain catastrophizing scale. Eur J Pain. (2020) 24(7):1228–41. 10.1002/ejp.1587 32416018

[43] Spielberger C Gorsuch R Lushene R Vagg PR Jacobs G. Manual for the State-Trait Anxiety Inventory (Form Y1—Y2). (1983).

[44] Gauthier J Bouchard S. Adaptation canadienne-française de la forme révisée du State–trait anxiety inventory de spielberger. [A French-Canadian adaptation of the revised version of Spielberger’s State–trait anxiety inventory]. Canadian J Behav Sci Revue Canadienne des Sci du Comportement. (1993) 25(4):559–78. 10.1037/h0078881

[45] Barnes LLB Harp D Jung WS. Reliability generalization of scores on the spielberger state-trait anxiety inventory. Educ Psychol Meas. (2002) 62(4):603–18. 10.1177/0013164402062004005

[46] Schubert T Friedmann F Regenbrecht H. The experience of presence: factor analytic insights. Presence. (2001) 10:266–81. 10.1162/105474601300343603

[47] Panahi-Shahri M. Reliability and validity of igroup presence questionnaire (IPQ). Int J Behav Sci. (2009) 3(1):27–34.

[48] Henri C Marchand S Giguère C Léonard G Potvin S. Inter-subject variability of pleasant pain relief using a data-driven approach in healthy volunteers. Front Pain Res. (2022) 3:1003237. 10.3389/fpain.2022.1003237 PMC9720129 36478768

[49] Borenstein M Hedges L Higgins JPT Rothstein HR. Comprehensive meta-analysis (version 2.2.027) [computer software]. Wiley (2005). p. 188–91.

[50] Stankewitz A Mayr A Irving S Witkovsky V Schulz E. Pain and the emotional brain: pain-related cortical processes are better reflected by affective evaluation than by cognitive evaluation. Sci Rep. (2023) 13(1):8273. 10.1038/s41598-023-35294-2 37217563 PMC10202916

[51] Miron D Duncan GH Catherine Bushnell M. Effects of attention on the intensity and unpleasantness of thermal pain. Pain. (1989) 39(3):345–52. 10.1016/0304-3959(89)90048-1 2616184

[52] Price DD. Psychological and neural mechanisms of the affective dimension of pain. Science. (2000) 288(5472):1769–72. 10.1126/science.288.5472.1769 10846154

[53] Price DD Harkins SW Baker C. Sensory-affective relationships among different types of clinical and experimental pain. Pain. (1987) 28(3):297–307. 10.1016/0304-3959(87)90065-0 2952934

[54] Rainville P Bt C Hofbauer RK Bushnell MC Duncan GH. Dissociation of sensory and affective dimensions of pain using hypnotic modulation. Pain. (1999) 82(2):159–71. 10.1016/S0304-3959(99)00048-2 10467921

[55] Rainville P Feine JS Bushnell MC Duncan GH. A psychophysical comparison of sensory and affective responses to four modalities of experimental pain. Somatosens Mot Res. (1992) 9(4):265–77. 10.3109/08990229209144776 1492527

[56] Dabu-Bondoc S Vadivelu N Benson J Perret D Kain ZN. Hemispheric synchronized sounds and perioperative analgesic requirements. Anesth Analg. (2010) 110(1):208–10. 10.1213/ANE.0b013e3181bea424 19861358

[57] Ecsy K Jones AK Brown CA. Alpha-range visual and auditory stimulation reduces the perception of pain. Eur J Pain. (2017) 21(3):562–72. 10.1002/ejp.960 27807916

[58] Zampi DD. Efficacy of theta binaural beats for the treatment of chronic pain. Altern Ther Health Med. (2016) 22(1):32–8. 26773319

[59] Arendsen LJ Henshaw J Brown CA Sivan M Taylor JR Trujillo-Barreto NJ Entraining alpha activity using visual stimulation in patients with chronic musculoskeletal pain: a feasibility study. Front Neurosci. (2020) 14. 10.3389/fnins.2020.00828 32973429 PMC7468433

[60] Gershon J Zimand E Pickering M Rothbaum BO Hodges L. A pilot and feasibility study of virtual reality as a distraction for children with cancer. J Am Acad Child Adolesc Psychiatry. (2004) 43(10):1243–9. 10.1097/01.chi.0000135621.23145.05 15381891

[61] Gold J Reger G Rizzo A Buckwalter G Kim S Joseph M. Virtual reality in outpatient phlebotomy: evaluating pediatric pain distraction during blood draw. J Pain. (2005) 6(3, Supplement):S57. 10.1016/j.jpain.2005.01.224

[62] Wolitzky K Fivush R Zimand E Hodges L Rothbaum BO. Effectiveness of virtual reality distraction during a painful medical procedure in pediatric oncology patients. Psychol Health. (2005) 20(6):817–24. 10.1080/14768320500143339

[63] Tashjian VC Mosadeghi S Howard AR Lopez M Dupuy T Reid M Virtual reality for management of pain in hospitalized patients: results of a controlled trial. JMIR Ment Health. (2017) 4(1):e9. 10.2196/mental.7387 28356241 PMC5390112

[64] Tanja-Dijkstra K Pahl S White MP Auvray M Stone RJ Andrade J The soothing sea: a virtual coastal walk can reduce experienced and recollected pain. Environ Behav. (2018) 50(6):599–625. 10.1177/0013916517710077 29899576 PMC5992839

[65] Bani Mohammad E Ahmad M. Virtual reality as a distraction technique for pain and anxiety among patients with breast cancer: a randomized control trial. Palliat Support Care. (2019) 17(1):29–34. 10.1017/S1478951518000639 30198451

[66] Tappe-Theodor A Kuner R. A common ground for pain and depression. Nat Neurosci. (2019) 22(10):1612–4. 10.1038/s41593-019-0499-8 31455879

[67] Thompson JM Neugebauer V. Cortico-limbic pain mechanisms. Neurosci Lett. (2019) 702:15–23. 10.1016/j.neulet.2018.11.037 30503916 PMC6520155

[68] King S Chambers CT Huguet A MacNevin RC McGrath PJ Parker L The epidemiology of chronic pain in children and adolescents revisited: a systematic review. Pain. (2011) 152(12):2729–38. 10.1016/j.pain.2011.07.016 22078064

[69] Abramowitz JS Deacon BJ Valentiner DP. The short health anxiety inventory: psychometric properties and construct validity in a non-clinical sample. Cognit Ther Res. (2007) 31(6):871–83. 10.1007/s10608-006-9058-1 32214558 PMC7088052

[70] Bjurstrom MF Irwin MR. Polysomnographic characteristics in nonmalignant chronic pain populations: a review of controlled studies. Sleep Med Rev. (2016) 26:74–86. 10.1016/j.smrv.2015.03.004 26140866 PMC4598249

[71] Minns S Levihn-Coon A Carl E Smits JAJ Miller W Howard D Immersive 3D exposure-based treatment for spider fear: a randomized controlled trial. J Anxiety Disord. (2018) 58:1–7. 10.1016/j.janxdis.2018.05.006 29909286

[72] Spiegel B Fuller G Lopez M Dupuy T Noah B Howard A Virtual reality for management of pain in hospitalized patients: a randomized comparative effectiveness trial. PLoS One. (2019) 14(8):e0219115. 10.1371/journal.pone.0219115 31412029 PMC6693733

[73] Flynn RM Richert RA. Cognitive, not physical, engagement in video gaming influences executive functioning. J Cogn Dev. (2018) 19(1):1–20. 10.1080/15248372.2017.1419246

[74] Sharek D Wiebe E. Measuring video game engagement through the cognitive and affective dimensions. Simul Gaming. (2014) 45(4–5):569–92. 10.1177/1046878114554176

[75] Sousa CV Hwang J Cabrera-Perez R Fernandez A Misawa A Newhook K Active video games in fully immersive virtual reality elicit moderate-to-vigorous physical activity and improve cognitive performance in sedentary college students. J Sport Health Sci. (2022) 11(2):164–71. 10.1016/j.jshs.2021.05.002 34004390 PMC9068577

