# Supplementary material for: The impact of an immersive digital therapeutic tool on experimental pain: a pilot randomized within-subject experiment with an active control condition
Source: Front Pain Res (Lausanne). 2024 Jun 6;5:1366892. doi: 10.3389/fpain.2024.1366892 (PMC11187308; doi:10.3389/fpain.2024.1366892)
Supplement: Supplementary file 1 [file Datasheet1.docx]

**The impact of an immersive digital therapeutic tool on pain perception:**

**An experimental study with an active control condition**

*Inhibitory conditioned pain modulation (iCPM)*

The efficacy of inhibitory CPM was measured in a separate experimental session ~one week apart from the VR session. To measure the inhibitory CPM, a continuous heat simulation was administered with a thermode for 2 minutes on the left forearm of participants (as described in section “Acute analgesic effect”). During thermal stimulation (test-stimulus), pain intensity was measured using a computerized visual analog scale, which ranged from 0 (no pain) to 100 (most intense pain tolerable). To capture the effects of inhibitory CPM, the test stimulus was administered twice, separated by the administration of the cold-pressor test (CPT) as the conditioning stimulus. The CPT consisted of the immersion of the opposite arm (right arm) into a bath of ice water that was kept constant at 10°C, for a maximum of 2 minutes, by continuously recirculating the water (Julabo F33-HL Heating/refrigerated circulator). The temperature was chosen to be painful enough to elicit the endogenous analgesia effect yet tolerable for 2 minutes (Bitar & al., 2018; Coulombe-Lévêque & al., 2021; Henri & al., 2022). By measuring pain perception (using the test stimulus) before and after the conditioning stimulus, it was possible to measure iCPM, which was defined as the reduction in pain perception observed between both administrations of the test stimulus (before and after the conditioning stimulus).

**References**

Bitar, N., Marchand, S., & Potvin, S. (2018). Pleasant Pain Relief and Inhibitory Conditioned Pain Modulation: A Psychophysical Study. *Pain Research and Management*, *2018*, 1935056. <https://doi.org/10.1155/2018/1935056>

Coulombe-Lévêque, A., Tousignant-Laflamme, Y., Léonard, G., & Marchand, S. (2021). The effect of conditioning stimulus intensity on conditioned pain modulation (CPM) hypoalgesia. *Canadian Journal of Pain*, *5*(1), 22-29. <https://doi.org/10.1080/24740527.2020.1855972>

Henri, C., Marchand, S., Giguère, C., Léonard, G., & Potvin, S. (2022). Inter-subject variability of pleasant pain relief using a data-driven approach in healthy volunteers. *Front Pain Res (Lausanne)*, *3*, 1003237. <https://doi.org/10.3389/fpain.2022.1003237>
